# PUBLISHING IN INTERNATIONAL JOURNALS AND THE EXPECTATION IT PLACES ON OUR WRITING: TIPS FROM AGEING & SOCIETY

**DOI:** 10.1093/geroni/igac059.308

**Published:** 2022-12-20

**Authors:** Sandra Torres

**Affiliations:** Uppsala University, Sweden, Uppsala, Uppsala Lan, Sweden

## Abstract

Understanding the differences between academic writing genres is crucial to nailing down the craft that is writing for peer-review journal publication. Based on the experience of Ageing & Society, this presentation will bring attention to the different readers that peer-reviewed journals cater to and how these differences impact how we craft manuscripts about the research we conduct. Using this as a starting point, as well as allusions to how data collected in specific national settings can be reported so that it can be used to contribute to ongoing international debates in social gerontology, this presentation offers the Editor-in-Chief’s top 10 tips for publishing in international peer-reviewed journals. By bringing specific attention to the routines that Ageing & Society utilize, and the expectations that its editors place on the different sections of a manuscript, this presentation aims to give attendees valuable insights into how to write for peer-reviewed outlets.

